# Bovine alkaline phosphatase in health and disease: A bibliometric and narrative review

**DOI:** 10.1016/j.vas.2026.100727

**Published:** 2026-06-04

**Authors:** Daniya Smagulova, Charlotte Dawson, Guilherme de Padua Nogueira, Michael J. D’Occhio, Laercio R. Porto-Neto, Isabella C. de Castro Lipp, Marina R.S. Fortes

**Affiliations:** a West Kazakhstan Marat Ospanov Medical University, Scientific and Practical Center, Maresyev 68, 030019, Aktobe, Kazakhstan; b The University of Queensland, School of Chemistry and Molecular Biosciences, St Lucia Campus, Qld 4072, Australia; c UNESP, Araçatuba, SP, Brazil; d The University of Sydney, School of Life and Environmental Sciences, Sydney, Australia; f Commonwealth Scientific and Industrial Research Organisation, Agriculture and Food, Queensland Bioscience Precinct, St Lucia, Queensland, Australia; g Southern Cross University, Faculty of Science and Engineering, Lismore, NSW 2480, Australia

**Keywords:** Beef cattle, Biomarkers, Dairy cattle, Enzymes, Isoforms, Isozymes

## Abstract

Alkaline phosphatases (ALPs) are enzymes involved in bone mineralisation, liver function, and intestinal health. In cattle, the activity of ALPs varies with age, nutrition, and physiological or pathological status. Despite decades of research, the understanding of isozyme-specific roles and diagnostic applications remains fragmented. This bibliometric and narrative review synthesised 473 peer-reviewed studies that measured ALPs in cattle, published between 1948 and 2025, retrieved from the Scopus database. Bibliometric analysis revealed a marked increase in publications over the past decade, with most studies focused on dairy cattle during pregnancy, lactation, and metabolic adaptation. Bone-associated ALP is consistently associated with skeletal growth and mineral metabolism, whereas hepatic- and placental-associated ALPs are linked to metabolic stress and reproductive processes. Altered activity of ALPs in serum or milk has been reported in conditions such as mastitis, milk fever, ketosis, parasitic infections, and respiratory disease, supporting its potential as a biomarker of health and disease. However, major limitations remain across the literature, including the underrepresentation of beef cattle and the frequent lack of isozyme and isoform differentiation. These limitations constrain biological interpretation and clinical applicability of measuring ALPs. Overall, ALPs have potential as tools for monitoring cattle health, supporting nutritional management and veterinary care. Future research should prioritise isozyme and isoform specific analyses and expand investigations across diverse breeds and production systems to improve the diagnostic utility of ALPs and drive its potential use in cattle systems.

## Highlights

• ALPs are key enzymes, linked to bone growth, liver function, and health in cattle.
• Activity of ALPs varies by age, diet, reproduction, and health.
• Dairy cattle studies dominate the current literature; beef cattle remain understudied.
• US, India and China lead in publication numbers, while Italy and Iran published high impact articles.
• Further studies dissecting isozymes and isoforms of ALPs are needed to develop ALPs as biomarkers for growth, metabolism, and disease across cattle breeds.

## Graphical abstract

**Figure:**
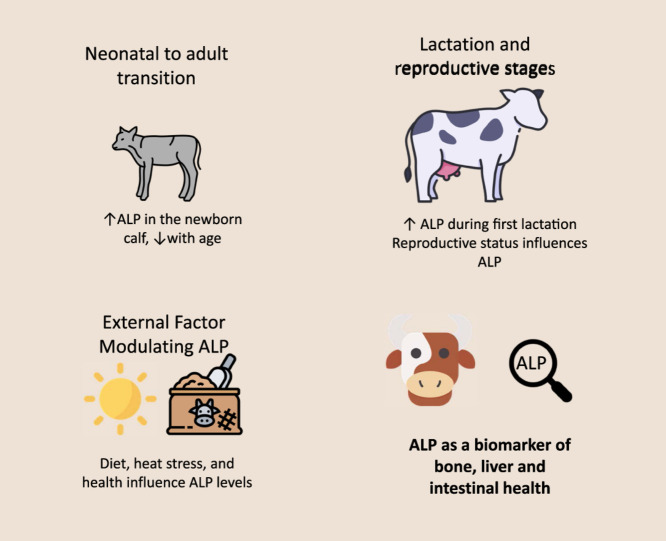

## Introduction

Alkaline phosphatases (ALPs) are a family of enzymes that hydrolyse phosphate esters, releasing inorganic phosphate (Pi) and thereby contributing to multiple physiological processes (Sharma et al., 2014). Through this activity, ALPs play a central role in regulating Pi bioavailability, which is indispensable for cellular metabolism and skeletal mineralisation. Pi serves as a structural component of nucleic acids and membranes and functions as a key regulator of intracellular signalling and protein activity. The bioavailability of Pi in circulation is maintained through the coordinated interplay of intestinal absorption, bone storage and release, and renal reabsorption (Choi, 2008). This interplay is orchestrated by ALPs. Therefore, ALPs are enzymes that provide valuable physiological insights in mammals, including cattle.

The activity of ALPs in serum is primarily derived from tissue-specific isoforms, most notably those associated with liver and bone. These enzymes have a relatively long half-life and are widely used to assess liver function and bone metabolism, although circulating ALP activity is also influenced by enzyme clearance (Levitt et al., 2022). In mammals, ALPs comprise a family of enzymes encoded by distinct genes, including the tissue-nonspecific ALP (TNAP), encoded by the *ALPL* gene, as well as intestinal (ALPI), placental (ALPP), and germ cell-associated (ALPG) forms. Importantly, the liver, bone, and kidney forms are not separate isozymes but represent tissue-specific isoforms of the TNAP enzyme encoded by *ALPL*, arising from post-translational modification and differential expression across tissues. In contrast, intestinal- and placental-associated ALPs are encoded by distinct genes and therefore represent true isozymes. This distinction is critical for accurate biological and clinical interpretation. For example, elevated bone-associated ALP (BALP) reflects osteoblastic activity and skeletal growth, whereas increased hepatic ALP is associated with hepatobiliary function and metabolic adaptation (Makris et al., 2023; Sato et al., 2005). Therefore, distinguishing between gene-derived isozymes and tissue-specific isoforms is essential when interpreting activity of ALPs in bovine physiology and pathology.

Multiple studies highlight the involvement of ALPs in growth, pregnancy, lactation, and phosphorus metabolism (Alfaro et al., 2024; Farman et al., 2024; Teneva et al., 2020). ALPs have a critical role in skeletal mineralisation and bone turnover, hepatic adaptation and biliary function, placental phosphate transfer and calcium homeostasis in mid–late gestation, intestinal barrier function and neonatal gut maturation, renal phosphate handling, and the metabolic adjustments of pregnancy and lactation. Many studies discuss ALPs in general, but some explored tissue-specific functions of ALPs. BALP, historically referred to as ALP3, is closely linked to bone turnover and mineralisation. Bone remodelling is essential for skeletal integrity, relying on the balance between osteoblast-driven bone formation and osteoclast-mediated bone resorption (Haarhaus et al., 2022). BALP reflects osteoblastic activity and is a reliable biomarker of skeletal health (Szulc et al., 2021). The practical value of BALP has been demonstrated in detecting defective bone mineralisation in beef cows caused by dietary phosphorus deficiency, which underscores its potential as an indicator of nutritional stress (Anderson et al., 2017). Further biochemical and functional studies on all ALPs isozymes and isoforms are required to fully understand and apply ALPs as a clinical or physiological biomarker in cattle.

In calves, serum activity of ALPs is markedly elevated at birth and declines within the first days of life (Asim & Al-Zaxoyi, 2023; Cattaneoet al., 2023; Hatate et al., 2016). However, ALPs levels remain higher in calves than in adults because of the rapid growth and skeletal development of young animals, and because colostrum intake may contribute to circulating ALPs activity (Asim & Al-Zaxoyi, 2023; Cattaneoet al., 2023; Hatate et al., 2016). BALP activity is elevated during the first 2 months of life, decreases immediately after weaning at around three months, peaks again near six months of age (likely associated with rapid skeletal growth) and then stabilises in mature animals, highlighting its association with skeletal growth and development in Holstein heifers that were weaned at 3 months of age (Chiba et al., 2020a). Further studies are required to describe the dynamics of BALP variation in early life stages, during rapid growth, in beef cattle breeds.

In relation to reproduction, ALPs levels rise during pregnancy and lactation. Placental-associated ALP contributes to calcium homeostasis in mid- and late gestation (Yokus & Cakir, 2006). Overall, the activity of ALPs increases after day 30 of gestation, stabilising towards parturition (Zhelavskyi et al., 2024). Higher activity of ALPs during lactation has been attributed to increased hepatic function and calcium mobilisation (Sato et al., 2005). In short, reference intervals for ALPs should consider gestation and lactation status.

Given its key physiological roles, ALPs have emerged as potential biomarkers for disease. Elevated levels of ALPs have been reported in milk and blood of cows with subclinical mastitis suggesting diagnostic potential for early detection (Yehia et al., 2023). ALPs and BALP are increased in cows with milk fever (Starič & Zadnik, 2010). ALPs levels in nasal secretions were proposed as a marker of respiratory disease (Ghazali et al., 2014). The cited studies highlight the potential diagnostic utility of ALPs for monitoring metabolic and infectious diseases in cattle. However, clinical thresholds to distinguish high activity of ALPs in healthy versus unhealthy lactation status are not yet available.

Although bovine ALPs have been widely investigated, existing studies are heterogeneous in their reported reference values, rarely distinguish between isozymes, and even less so between isoforms, and provide limited clinically defined thresholds, underscoring the need for a review to integrate the existing knowledge, identify gaps and better define ALPs potential for cattle husbandry and clinical diagnostics (Makris et al., 2023; Mohebbi et al., 2010; Mohri et al., 2007; Sato et al., 2005). This bibliometric and narrative review provides an integrated overview of research trends, physiological functions, and potential clinical applications of ALPs in cattle. This review focused on peer-reviewed papers that measured ALPs to integrate existing knowledge and identify gaps to guide future research.

## Materials and methods

### Search strategy

A comprehensive literature search was conducted from the Scopus database to assess and synthesise literature that measured ALPs in cattle. The search was designed to capture all relevant studies on measurements and measured activity of ALPs conducted in cattle. First, the primary keywords "cattle" and "alkaline phosphatase" were expanded with relevant synonyms using the MeSH (Medical Subject Headings) vocabulary thesaurus (Table 1). Next, all the collected keywords were entered into the Scopus database (https://www.scopus.com, accessed on 24th of April 2024, and repeated on 11th of February 2025). Relevant articles were identified by screening their occurrence in the Article Title, Abstract, and Keyword fields. The search was conducted without restrictions on publication date to capture the spectrum of research available on the topic.

**Table 1:** Search queries for the Scopus (Literature search date: 24 April 2024).

Number	Queries	Search result
#1	“Cattle” OR “Cow” OR “Cows” OR “*Bos indicus*” OR “*Bos indicus* Cattle” OR “*Bos indicus* Cattles” OR “Cattle, *Bos indicus*” OR “Cattles, *Bos indicus*” OR “Indicine Cattle” OR “Cattle, Indicine” OR “Cattles, Indicine” OR “Indicine Cattles” OR “Zebu” OR “Zebus” OR “Holstein Cow” OR “Cow, Holstein” OR “Dairy Cow” OR “Cow, Dairy” OR “Dairy Cows” OR “Beef Cow” OR “Beef Cows” OR “Cow, Beef” OR “Bos grunniens” OR “Yak” OR “Yaks” OR “*Bos taurus*” OR “Taurine Cattle” OR “Cattle, Taurine” OR “Cattles, Taurine” OR “Taurine Cattles” OR “Taurus Cattle” OR “Cattle, Taurus” OR “Cattles, Taurus” OR “Taurus Cattles” OR “Cow, Domestic” OR “Domestic Cow” OR “Domestic Cows”	545,595
#2	“Alkaline phosphatase”	187,911
#3	#1 AND #2	3540

**Fig. 1:**
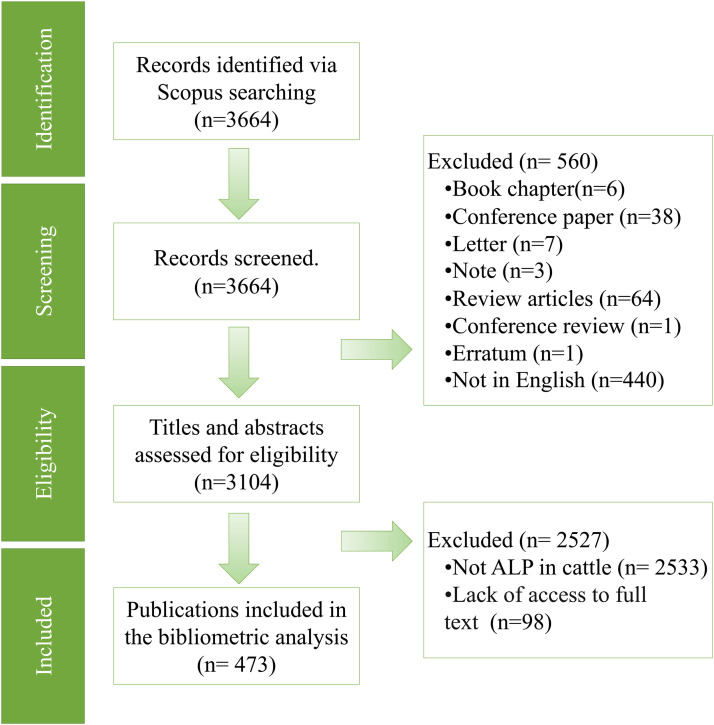
The flow diagram illustrates the screening process of the bibliometric analysis conducted to study ALPs in cattle.

To ensure the precision and pertinence of the included studies, particular inclusion and exclusion criteria were implemented (Fig. 1). The inclusion criteria had four considerations: 1. Peer-reviewed articles published in English. 2. Studies specifically focused on ALPs in cattle. 3. Studies focused on ALPs levels measured in beef and/or dairy cattle. 4. Studies where ALPs were measured as a biomarker and associated with health conditions in cattle. The exclusion criteria were: 1. Studies that did not involve cattle or focused on species other than cattle. 2. Review articles, book chapters, conference papers, letters, notes, conference reviews, and errata. 3. Studies without an accessible full text to verify measurements of ALPs (Fig. 1). A limitation of this methodology is that only articles published in English were included.

Screening and eligibility assessment were performed manually by a single author (D.S.) using the predefined inclusion and exclusion criteria. Records retrieved from Scopus were exported into R Studio for bibliometric analyses and subsequently into Microsoft Excel for data verification, thematic organisation, and narrative synthesis. No automated screening platforms or artificial intelligence-assisted selection tools were used. The study selection workflow and exclusion reasons are summarised in Fig. 1.

For this bibliometric and narrative review, we conducted a literature search of the Scopus database benefiting from its bibliometric framework (Baas et al., 2020). While this approach may have excluded studies indexed exclusively in other scientific platforms, it ensured consistency and reproducibility of the search strategy. Restricting the analysis to studies with accessible full texts that included measurements of ALPs prioritised methodological rigour and reliability of data interpretation, but we acknowledge that some information may not have been captured. Scopus methodological approach translated into a robust, accessible, evidence-based bibliometric review.

For the narrative synthesis, key information was manually extracted from the full text of each included study, including reported ALPs values and units, physiological or pathological context, cattle breed, production system (beef or dairy), age or physiological status, intervention or treatment where applicable, and the general type of ALPs measurement or biomarker application described. These data were manually organised thematically into Table 3, Table 4.

### Data visualisation

Scopus was chosen for its broad range of journals and efficient citation analysis (Falagas et al., 2008). Data from the retrieved literature were exported to BibTeX file format, including types of documents, languages, countries, authors, institutions, journals, and citations. Bibliometric analysis was conducted using the R package bibliometrix and its shiny web interface, biblioshiny, within R Studio software (Version 2023.12.1 + 402) (Aria & Cuccurullo, 2017). This library enabled analysis of the distribution of yearly published articles and average citations, core sources using Bradford’s law, author productivity, original articles cited globally, and frequency of keywords used between 1948 and 2025. The search was repeated on 11th February 2025 to verify previous findings and to include any new research articles relevant to the review.

## Results

The analysis of ALPs in cattle encompassed the examination of 208 scientific journals and 473 documents from 1948 to 2025 (Fig. 1). The study revealed an annual growth rate of 0.9%, with an average document age of 22.3 years and an average of 16.86 citations per document. The content analysis identified 2553 keywords and 1014 authors' keywords related to ALPs in cattle. In total, 1941 authors contributed to this topic, with 25 single-authored articles. There was an average of 4.87 co-authors per document, and international co-authorships accounted for 15.43% of collaborations.

### Extent of publications and source of research

**Fig. 2:**
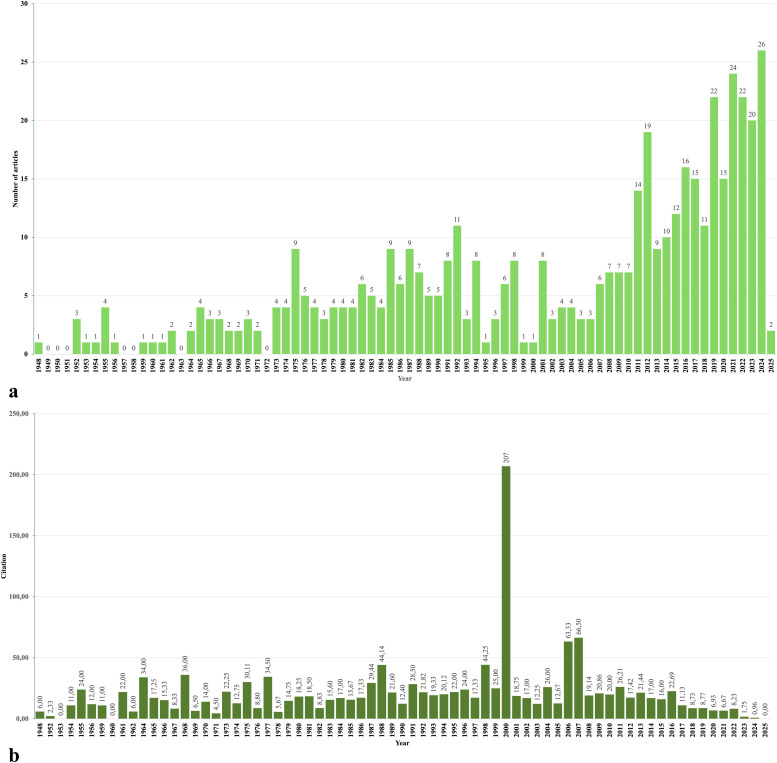
Cattle alkaline phosphatase research publication (**a**) and citation (**b**) per year.

Since 1948, the publication of research articles on cattle ALPs has fluctuated with distinct peaks and troughs observed over the decades. The years 2019, 2021, 2022, and 2024 were the most productive for publications (Fig. 2a) in this topic. The citation rate fluctuated over the reviewed period with a significant peak in 2000 (Fig. 2b).

An analysis utilising Bradford’s Law showed that 12 journals significantly contribute to the literature on ALPs in cattle, with the “Journal of Dairy Science” (JDS) as the most prolific contributing 46 articles. The prominence of JDS is attributed to its open-access policy, high Cite Score ranking in the field (14th out of 456 in 2022), and a Journal Impact Factor of 4.4, placing it in the 97th percentile in Agricultural and Biological Sciences (Table 2). This is also evidence for a bias towards research in dairy cattle as opposed to beef cattle. Most studies investigated Holstein animals.

**Table 2:** Identification of core journals connected with cattle ALPs by Bradford Law analysis (1948–2025).

Rank	Core journals	Articles (Frequency)
1	Journal of Dairy Science	46
2	Journal of Animal Science	22
3	Indian Journal of Animal Sciences	14
4	Comparative Clinical Pathology	11
5	Animals	9
6	Journal of Veterinary Medical Science	9
7	Research in Veterinary Science	9
8	Indian Journal of Animal Research	8
9	Journal of Biological Chemistry	8
10	Livestock Science	7
11	Veterinary World	7
12	Zentralblatt Für Veterinärmedizin Reihe A	7

### Analysis of productivity and impact by author and institution

**Fig. 3:**
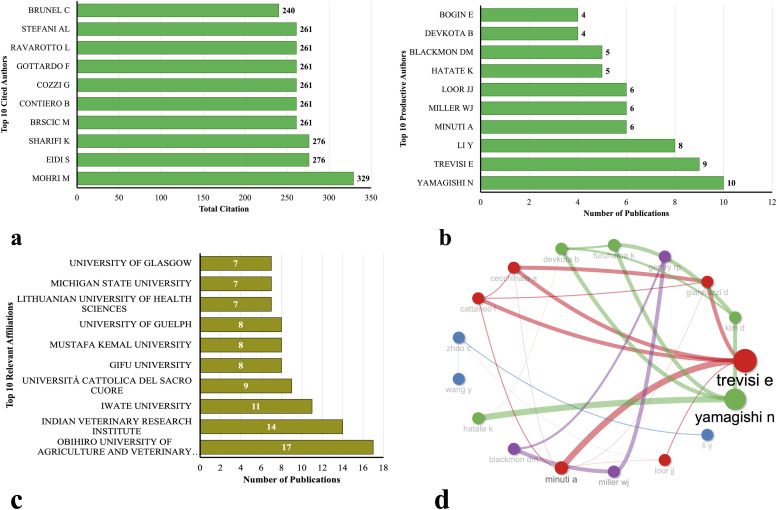
Productivity of author and institution: (a) Authors with the highest citation counts; (b) The number of articles of productive authors; (c) The ten most significant affiliations related to the research; (d) The network of collaborations among authors.

The productivity of authors was measured by the number of citations and research articles published (Fig. 3a, 3b). For the top 10 affiliations, Obihiro University of Agriculture and Veterinary Medicine had the most publications (n = 17) (Fig. 3c). The network of collaborations is shown in Fig. 3d.

### Analysis of scientific productivity by country

**Fig. 4:**
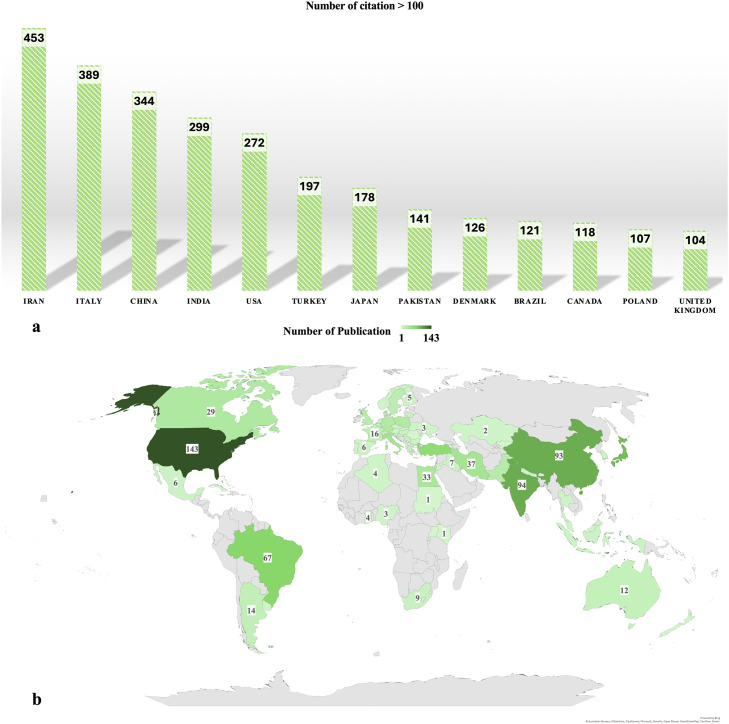
Country production and citation. (a) Mapped 13 countries’ citation rates (>100) and (b) the number of publications from different countries.

In total, 61 countries contributed to publishing ALPs research in cattle from 1948 to 2025. Distinct research productivity and impact patterns were observed by analysing the total citations and the frequency of published articles by country (Fig. 4). The United States, India, China, and Japan have a high frequency of publications. Iran and Italy have fewer publications (37 and 40, respectively) but a high number of total citations, suggesting that their research is highly impactful.

#### Analysis of keyword trends

**Fig. 5:**
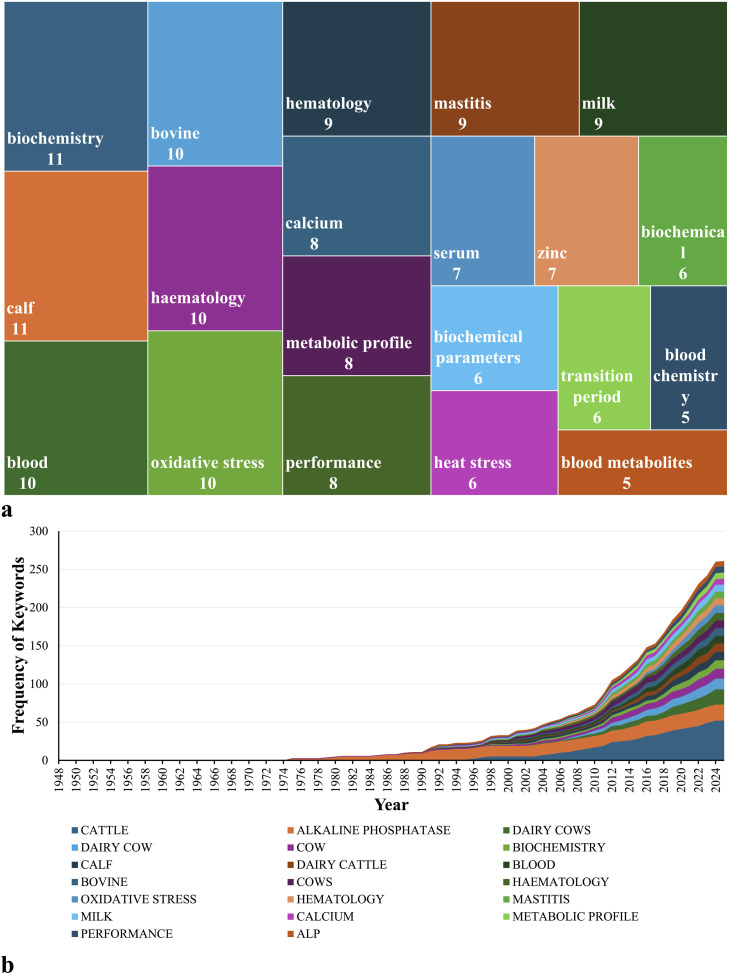
A TreeMap depicting the 20 most frequently used authors’ keywords (a) and the distribution of keywords associated with cattle and ALP-related articles over 76 years, starting from 1948 (b).

During the keyword analysis, the specific terms utilised in the search process (Table 1) were excluded from evaluation. A substantial proportion of studies investigating the association between activity of ALPs and cattle incorporate the keywords “biochemistry” and “calf”. To characterise the key enzymatic parameters, researchers frequently employed terms such as “blood”, “haematology”, “hematology”, “metabolic profile”, “serum”, “biochemical”, “biochemical parameters”, “blood chemistry”, and “blood metabolites” (Fig. 5a). A set of 20 frequently occurring keywords was identified. The use of these keywords has been documented since 1991 with a progressive increase in their prevalence over time (Fig. 5b). These keywords indicate that blood and serum samples are the primary biological materials collected in ALPs studies in cattle. Although less common, some studies also investigated the activity of ALPs in milk and nasal secretions.

### Reference intervals, pathological conditions, and coding genes

Reported ALPs values varied substantially across studies, with values for healthy adult cattle generally ranging between approximately 25 and 250 U/L, while markedly higher values were consistently observed in diseases (Table 3) and in neonatal or growing calves (Table 4). External variables, such as dietary supplementation, heat stress, and infectious diseases affect the activity of ALPs (Tabe 3), highlighting how nutrition, environment, and health status can modulate enzyme levels and their interpretation. This review compiles reported ALPs values and reference intervals from the literature together with the physiological, pathological, and methodological contexts in which they were measured, including breed, age, production system, health status, and assay used (Table 4). We also summarise the genomic annotation of bovine ALPs genes, along with their transcripts, and protein IDs to support discussion of isozyme and isoform differentiation (Table 5). The compiled and organized literature provides the thematic foundation for the narrative synthesis of ALPs biological functions in cattle, presented in the following Discussion section.

**Table 3:** Influence of external factors on ALPs levels in cattle: diets, heat stress, and infections.

Variable	Intervention	Breed	Age	Assay method / kit	Effect on ALPs levels	Findings	Reference
Dietary Interventions	Zn supplementation	*Bos indicus* × *Bos taurus*	2yo	Diagnostic kits (Glaxo)	↑ (max +131.38 KAU.100 mL⁻¹)	The significantly higher levels of alkaline in the seminal plasma of Zn-supplemented groups compared to the control may be attributed to their nature as Zn-dependent metalloenzymes, requiring Zn ions both for *catalytic function and structural stability.*	Kumar et al., 2006
	Zn-methionine supplementation	Holstein	NA	Commercial ELISA kits (Biosino Biotechnology Co. Ltd., Beijing, China)	↑ (max +9.2 U/L at 40 mg/kg DM)	ALP activity increased in cows receiving *40mg or more of Zn/kg of DM*, indicating that Zn-Met supplementation may enhance the function of this zinc-dependent enzyme.	Chen et al., 2020
	Anion-rich diet	Friesian- Holstein × Holstein-Friesian	6.7 + 0.8yo	Test combination (Beckman Synchron CX ∼systems, Mijdrecht, The Netherlands) and a computerized auto-analyser (Beckman Synchron CX5, Mijdrecht, the Netherlands)	↔	*No effect* on plasma ALPs levels or *bone formation* despite increased Ca excretion.	Schonewille et al., 1994
	Low-phosphorus diet (0.16–0.19% P DM)	Xia-Nan (crossbred)	NA	Commercial kit (Nanjing Jiancheng), DG5033A microplate reader	↑ (max +26.2 U/L vs control at 0.25% P)	Serum ALPs increased with decreasing dietary P, reflecting compensatory bone metabolism and Ca-P homeostasis under hypophosphatemia (P = 0.006, linear trend P = 0.002).	Song et al., 2025
Heat stress	High HLI	Holstein-Friesian	NA	An automatic clinical chemistry analyser (Cobas 8000 Modular Analyser; Roche Diagnostics, Indianapolis, IN, USA)	↓ (No Shade:0.15 U/L per unit HLI, *P* < 0.0001); ↔ (With Shade:0.01 U/L per unit HLI, *P* = 0.87).	Providing shade helps *mitigate the reduction in plasma ALPs* levels caused by high environmental temperatures in dairy cows.	Tuyttens et al., 2015
	Summer season	NA	NA	Kinetic analysis using commercial kits (Instrumentation Laboratory, Lexington, MA).	↓ (−10.74 U/L vs. winter; −5.58 U/L vs. spring)	The marked reduction in ALPs levels during summer (46.09 U/L), in contrast to higher values in winter (56.83 U/L) and spring (51.67 U/L), suggests that *heat stress in the summer season has a suppressive effect on ALPs activity*.	Calamari et al., 2018
	Heat stress + dietary modifications (fermentability, grain, oils)	Holstein	NA	An autoanalyzer (Abbott Alycon 300, USA) using commercial kits (Pars Azmoon Co., Tehran, Iran)	↑ (linear: +19.8 U/L, from 65.1 to 84.9 U/L)	In multiparous lactating dairy cows, increased blood ALPs levels alongside reduced MDA concentrations indicate that enhanced diet fermentability may *alleviate oxidative stress*; however, supplementation with fish oil has been linked to elevated circulating ALP, suggesting a potential association with *partial liver dysfunction.*	Kargar et al., 2015; Nasrollahi et al., 2019;
	Moderate to severe heat stress (THI > 80)	Holstein-Friesian	3–5 years	Rayto Chemray 120 spectrophotometer, Randox kits	↔ (no significant change, p = 0.653)	ALP was not affected by heat stress, unlike many other metabolic, endocrine, and inflammatory parameters (eHsp70, TNFα, cortisol, T3, T4, NEFA, glucose, etc.).	Blond et al., 2024
Infections	*Theileria annulata*	Holstein Friesian-Sahiwal	1week −1 month of age	An autoanalyzer (Beckman Clinical System-700) using autokits.	↑ (no absolute values reported)	The significant rise in ALPs from day 16 onward suggests that only parasitaemia causes notable biochemical changes, with elevated ALP—abundant in muscle and liver—indicating *tissue necrosis or disease.*	Sandhu et al., 1998
		Different breeds	1–7 yo	VetScan brand (ABAXIS USA) biochemistry analyser and its kits	↑ (+58.58 U/L pre-treatment vs. control);	ALP level was *111.40U/L before treatment,* and 64.18 U/L after treatment.	Dede et al., 2014
		Cross-bred	NA	Not specified	↑ (357.67 ± 32.58 IU/L in infected vs 176.94 ± 130.9 IU/L in uninfected)	Significant elevation of ALPs in T. annulata-positive animals, indicating hepatic injury	Suryawanshi et al., 2024
	*Babesia* spp.	NA	NA	Colorimetric, microprocessor-based colorimeter; Accurex Biomedical Pvt. Ltd., India	↑ (+264.33 U/L vs. control, *P* < 0.001)	A significant increase in the ALPs level in *Babesia* spp. infected cattle (441.68 U/L) compared to non-infected (177.35 U/L) was observed, associated with *hepatic injury or dysfunction*, indicating the harmful effect of toxic metabolites on liver cells.	Ghimire et al., 2022
	*Trichostrongylus colubriformis*	Holstein or Holstein-Angus	8 weeks of age	Histochemical	↓ (progressive - no absolute values reported)	ALP activity was highest in the proximal small intestine of noninfected calves but progressively declined during T. colubriformis infection, corresponding with *worsening intestinal damage.*	Shayo & Benz, 1979

Note: KAU – Karmen units; DM – dry matter; HLI – Heat Load Index; MDA – malondialdehyde.

**Table 4:** Reported reference intervals and values of serum alkaline phosphatase (ALP) activity in cattle across different ages, physiological groups, management systems, and pathological conditions compiled from published studies and institutional diagnostic laboratories.

Breed / Type	Age / Parity	Total ALPs (U/L)	Bone ALPs (U/L)	Liver ALPs (U/L)	Method	Clinical / Research Notes	Source
NEONATAL AND PRE-WEANED CALVES							
Holstein / dairy	1–14 days	29 - 187			Not specified	Established foundational age-specific Ris for cattle; emphasises the decline of ALPs with age.	Lumsden et al., 1980
	24–48 h	approx. 780			Commercial kit (Pars Azmoon, Tehran, Iran), an autoanalyzer (Biotecnica, Targa 3000, Rome, Italy)	ALP is highly dynamic in young calves; it supports the need for age-adjusted Ris in diagnostics.	Mohri et al., 2007
	14 days	approx. 290					
	28 days	approx. 260					
	42 days	approx. 380					
	56 days	approx. 410					
	70 days	approx. 550					
	84 days	approx. 460					
	40 days	419			Commercial kit (Boehringer Mannheim), Automated analyser (Hitachi 705)	Confirms limited diurnal variation; strengthens reliability of ALPs measurement in practice.	Dubreuil & Lapierre, 1997
	60 days	509					
	80 days	442					
	100 days	416					
	<1 week	830.43 ± 321.07			Hitachi 7180, p-nitrophenyl phosphate	Newborn calves; high ALPs due to colostrum intake and bone growth	Lee et al., 2023
	1 month	666.70 ± 304.81				Rapid bone growth phase	
	3 months	664.08 ± 243.57					
	6 months	648.92 ± 236.22					
Shorthorn / beef	1 day	430.5			Automated analyser (Technicon SMA II system)	It shows that ALPs decline markedly, evidencing the importance of age-specific interpretation.	Doornenbal et al., 1988
	43 days	308.1					
	80 days	387.0					
	109 days	193.8					
	137 days	206.6					
	165 days	179.5					
	218 days (weaning)	135.9					
	247 days	196.0					
	275 days	236.7					
	303 days	220.0					
	331 days (yearling)	212.6					
Aberdeen Angus / beef	4 days	228.0			Biochemical, XT20i automatic analyser (Thermo Fisher Scientific, Finland)	Follows bulls to maturity and shows a decline in ALPs with age.	Pavlík et al., 2010
	56 days	242.4					
	95 days	217.8					
GROWING AND YOUNG CATTLE							
Holstein / dairy	2 weeks - 6 months	16 - 129			Not specified	Age-related decline continues (see neonatal section).	Lumsden et al., 1980
	6 months - 2 years	22 - 82					
	12 months	662.20 ± 194.26			Hitachi 7180, p-nitrophenyl phosphate	Heifers before first calving; still elevated ALPs due to growth	Lee et al., 2023
Crossbred	7.5 months (bulls)	173.6			Automated analyser (Technicon SMA II system)	Age-related decline persists.	Doornenbal et al., 1988
	7.5 months (steers)	168.2					
	12.5 months (bulls)	273.6					
	12.5 months (steers)	221.5					
Aberdeen Angus / beef	116 days	153.0			Biochemical, XT20i automatic analyser (Thermo Fisher Scientific, Finland)	Continues age-related decline	Pavlík et al., 2010
	155 days	175.2					
	168 days	133.8					
	207 days	130.2					
	252 days	154.2					
	291 days	136.8					
	329 days	128.4					
	368 days	161.4					
	434 days	141.0					
	473 days	150.6					
	525 days	123.0					
	564 days	119.4					
ADULT CATTLE							
Holstein / dairy	2 years+	3 - 46			Not specified	As described above in the *Neonatal and Pre-weaned Calves* section	Lumsden et al., 1980
	4.0 ± 1.3 years	71.1 – 258.9			Kinetic, nitrophenylphosphate reaction, Pentra C400 ISE analyser	New reference intervals for German Holstein Friesian cows; considers pregnancy and days in milk (DIM). ALPs negatively correlated with age and parity.	Theile et al., 2025
	Parous, mid-lactation cows	171.26 ± 55.06			Hitachi 7180, p-nitrophenyl phosphate	Adult cows; ALPs within adult reference range	Lee et al., 2023
Shorthorn / beef	2 years	122.7			Automated analyser (Technicon SMA II system)	Age-dependent changes continue.	Doornenbal et al., 1988
	4–5 years	120.6					
	6–10 years	179.4					
Crossbred	12–14 years	67.2 - 81.7					
Adult Cattle (RIs)	Adult	27 - 127			Cobas 501 chemistry analyser	Widely used clinical pathology range; method-dependent (auto-analyser).	Cornell AHDC (USA)
	Adult	25 - 127			Cobas 6000 c501	Modern lab Ris* emphasises instrument- and method-specificity for ALP.	University of Guelph (Canada)
LACTATION STAGE							
Holstein / dairy	Last 2 months lac. / Day 1	124.0			Commercial kit (Boehringer Mannheim), Automated analyser (Hitachi 705)	Reliability confirmed (see neonatal section).	Dubreuil & Lapierre, 1997
	Last 2 months lac. / Day 15	131.0					
	Last 2 months lac. / Day 29	116.0					
	Last 2 months lac. / Day 43	102.0					
	Last 2 months lac. / Day 57	106.0					
	Dry period	approx. 78	approx. 20	approx. 57	Commercial ALPs kit (Liquitech ALP, Roche Diagnostics Co., Ltd., Japan), Automatic analyser (Automatic Analyzer 7060, Hitachi, Ltd., Japan)	Bovine ALPs have limited specificity for hepatobiliary disease compared to dogs/cats.	Sato et al., 2005
	Early lactation (8–50 days)	approx. 120	approx. 34	approx. 80			
	Peak lactation (51–110 days)	approx. 110	approx. 35	approx. 75			
	Mid lactation (111–220 days)	approx. 100	approx. 34	approx. 73			
	Late lactation (221 days to dry)	approx. 90	approx. 31	approx. 62			
	Lactating, 5–540 DIM	54.17			ILAB 650 clinical auto-analyser (Instrumentation Laboratory)	Healthy cows; part of a GWAS for stress response biomarkers; heritability of ALPs = 0.46 (SE 0.071)	Passamonti et al., 2024
PARITY AND LACTATION							
Holstein (Primiparous) / dairy	Early lactation	approx. 260	approx. 160	approx. 78	Agarose gel electrophoresis system by Helena Laboratories (Saitama, Japan)	Primiparous cows, still undergoing growth, showed higher serum t-ALP and ALP3 activities, reflecting active skeletal development. In multiparous cows, the late lactation rise in ALP3 may indicate bone formation associated with declining milk yield.	Chiba et al., 2020
	Mid lactation	approx. 310	approx. 210	approx. 80			
	Late lactation	approx. 290	approx. 180	approx. 61			
Holstein (Multiparous) / dairy	Early lactation	approx. 160	approx. 75	approx. 73			
	Mid lactation	approx. 170	approx. 100	approx. 61			
	Late lactation	approx. 210	approx. 125	approx. 60			
PARITY EFFECT							
Holstein / dairy	Parity 1	115.19	45.12		Kinetic method using p-nitrophenylphosphate	The results show that enzyme activity decreases with increasing parity.	Mohebbi et al., 2010
	Parity 2	84.50	36.15				
	Parity 3	88.58	29.52				
	Parity 4	81.16	19.21				
	Parity 5	72.16	15.22				
PREGNANCY AND FARMING SYSTEM							
Mixed (Organic herd)	Non-pregnant	108.6			Bio-la- test (Lachema Brno, Catalogue No 1300,250) and Specol photometer (Carl Zeiss, Jena)	ALP activity was higher in the organic herd than in the conventional herd, with the highest values observed in cows pregnant for over 140 days in both groups.	Šoch et al., 2008
	First 140 days of pregnancy	160.2					
	>140 days of pregnancy	211.2					
	After calving	125.4					
Mixed (non-organic herd)	Non-pregnant	85.8					
	First 140 days of pregnancy	109.8					
	>140 days of pregnancy	120.0					
	After calving	94.2					
LONGITUDINAL STUDY							
Shorthorn / beef	On calving day	192.9			Automated analyzer (Technicon SMA II system)	Age-dependent changes continue.	Doornenbal et al., 1988
	43 days after calving	155.3					
	80 days after calving	120.9					
	109 days after calving	106.2					
	137 days after calving	135.2					
	165 days after calving	154.9					
	218 days after calving (weaning)	235.8					
	247 days after calving	203.7					
	275 days after calving	98.2					
	303 days after calving	135.3					
	331 days after calving (dry period)	130.3					
PATHOLOGICAL CONDITION							
MASTITIS							
Holstein / dairy	7–9 years, multiparous lactating	Control: 58.97 CMT +1: 125.94 CMT +2: 154.21 CMT +3: 195.21			Enzymatic kinetic, Spectrum diagnostic kit (Spectrum diagnostic, Egypt)	Serum ALPs significantly higher in all subclinical mastitis groups vs. subclinical mastitis -free (p < 0.05). CMT+3 higher than CMT+1 (p < 0.05).	Yehia et al., 2023
**CLINICAL KETOSIS**							
	<3 weeks postpartum, multiparous (parity 2–4)	Control: approx. 60 Clinically ketotic: approx. 140			Automatic biochemistry analyser (Sekisui Medical Co. Ltd.); enzymatic method; commercial ALPs kit (Randox Laboratories, cat. no. AP502)	Serum activity of ALPs was markedly greater in cows with clinical ketosis (beta-hydroxybutyrate >3.0 mM) compared to healthy controls (beta-hydroxybutyrate<0.6 mM).	Shi et al., 2021
RETAINED PLACENTA							
Holstein–Friesian / dairy	Multiparous, at 24 h postpartum	Control: 84.5 Retained placenta: 118.4			Automatic biochemical analyser (ERBA XL 600); colorimetric method; commercial kits	Elevated ALPs (p < 0.05) may originate from placental tissue rather than liver injury, as other liver markers (alanine aminotransferase, aspartate aminotransferase, albumin) did not differ between groups.	Li et al., 2021
PYOMETRA							
Holstein-Friesian / dairy	5–7 years, parity 3–5	Control: approx. 60 Pyometra: approx. 85			Automatic clinical biochemistry analyzer (ERBA CHEM-7); colorimetric method; commercial kits (Biodiagnostic)	ALP significantly increased in cows with pyometra compared to healthy controls (p < 0.05). Other liver enzymes (alanine aminotransferase, aspartate aminotransferase) remained within normal range.	Amin et al., 2021
HYPOZINCEMIA							
Holstein / dairy	<1 month of age	Control: 81.26 Hypozincemic: 66.16 Treated: 64.52			Calorimetric method; commercial kits (Elitech (Egypt))	Treatment with oral zinc oxide improved clinical signs but ALPs remained lower than controls, possibly due to continued bone growth demands. (p < 0.01 for diseased vs. control)	El-Maghraby & Mahmoud, 2021
HYPOCALCEMIA							
Simmental / dairy	5.5years, mixed parity	Cut-off 52.5 U/L			Cobas C111	Antepartum ALPs to predict hypocalcaemia (Ca < 2 mmol/L) in herds with high prevalence. Sensitivity 90%, Specificity 91%, AUC 0.929.	Arnold et al., 2024
Simmental, Holstein-Friesian / dairy		Cut-off 81.5 U/L				Antepartum ALPs to predict hypocalcaemia in randomly selected herds. Sensitivity 73%, Specificity 63%, AUC 0.683.	

Note: Ris - Reference intervals; CMT - ***California Mastitis Test;*** AUC – Area Under the Curve.

**Table 5:** Symbol and position for ALPs’ genes, with their transcript IDs, protein IDs, and chromosomal positions (on chromosome 2). Gene annotation data for ALPs retrieved from Ensembl via BioMart (Ensembl Release 115 (September 2025)).

Gene symbol	Transcript stable ID	Transcript name	Gene start (bp)	Gene end (bp)	Protein stable ID
*ALPL*	ENSBTAT00000011783	ALPL-202	2:131,177,554	2:131,233,641	ENSBTAP00000011783
*ALPL*	ENSBTAT00000071091	ALPL-201	2:131,177,554	2:131,233,641	ENSBTAP00000063499
*ALPL*	ENSBTAT00000066596	ALPL-203	2:131,177,554	2:131,233,641	ENSBTAP00000065772
*ALPI*	ENSBTAT00000005937	ALPI-204	2:120,096,973	2:120,106,454	ENSBTAP00000005937
*ALPI*	ENSBTAT00000043359	ALPI-203	2:120,096,973	2:120,106,454	ENSBTAP00000040934
*ALPI*	ENSBTAT00000084059	ALPI-201	2:120,096,973	2:120,106,454	ENSBTAP00000063305
*ALPI*	ENSBTAT00000073739	ALPI-202	2:120,096,973	2:120,106,454	ENSBTAP00000069504

## Discussion

Reviewed studies indicate significant progress but also gaps in understanding on the role of ALPs in cattle biology, as well as its practical relevance for veterinary medicine and livestock production systems. Insights from this bibliometric and narrative review underpin the following discussion on ALPs isozymes and isoforms, the implications of ALPs as biomarkers for veterinary practice, and help to identify areas for further research to address gaps in knowledge.

### ALP isozymes and isoforms

Specific isozymes and isoforms of ALPs have markedly different and often tissue- or organ-specific roles in mammals. Two separate genes encode the major annotated bovine ALPs isozymes: TNAP (encoded by the *ALPL* gene) and the intestinal-associated ALP (encoded by *ALPI*). The placental-associated ALP gene, *ALPP*, and germ-cell/placental-like ALP gene, *ALPG,* are clearly annotated in many mammalian genomes but were not clearly identified in the bovine Ensembl annotation used herein Table 5, despite being mentioned in cattle studies. Within *ALPL*, post-translational modifications generate distinct isoforms associated with bone, liver, and kidney tissues. While some studies differentiate among specific isozymes or isoforms, others report only total TNAP activity.

Historically, electrophoretic studies in veterinary biochemistry frequently referred to bone-, liver-, and intestinal-associated ALP fractions as ‘isozymes’ based on electrophoretic mobility patterns. For example, BALP was known as ALP3 (Chiba et al., 2020). In the present review, we distinguish between true gene-derived isozymes and tissue-associated isoforms encoded by the same TNAP gene (*ALPL*). This distinction is proposed to help standardize ALP terminology and is used throughout this review whenever possible. However, many studies reported only total ALP activity.

### Bone-associated ALP activity

BALP is a central marker of bone metabolism reflecting active osteoblast function and participating directly in mineralisation. Its enzymatic activity promotes the hydrolysis of pyrophosphate, a potent inhibitor of mineral deposition, which facilitates matrix formation and mineralisation (Felix & Fleisch, 1974).

Reproductive physiology highlights BALP’s clinical significance. Serum BALP levels increase around parturition indicating enhanced bone turnover and osteoblast activity. BALP declines shortly after calving in both primiparous and multiparous cows due to the combined stress of parturition and the heightened calcium demands of lactation (Kim et al., 2010; Kurosaki et al., 2007). BALP concentrations are consistently higher in primiparous cows than in multiparous groups likely reflecting the ongoing skeletal growth and greater osteoblastic activity of younger animals (Kurosaki et al., 2007; Meyer & Harvey, 2004). During early lactation, bone resorption predominates without a parallel increase in bone formation resulting in relatively stable BALP levels (Filipović et al., 2008). BALP activity decreases with increasing parity in dairy cows highlighting its value as a practical indicator of age-related bone metabolism and calcium status (Mohebbi et al., 2010).

Endocrine and nutritional factors also modulate BALP activity. Prolonged adrenocorticotropic hormone infusion elevates cortisol which suppresses mineral homeostasis and reduces BALP activity measured as U/L (Kim et al., 2012). Dietary interventions provide evidence that links calcium and vitamin D supplementation to altered activity of ALPs supporting its value as a marker of bone metabolism beyond hepatic function (Silva et al., 2021; Singh et al., 2021; Ruiz-González et al., 2023). However, not all interventions elicit consistent responses. Calcitriol (vitamin D) administration in nonpregnant, nonlactating Holstein cows increased osteocalcin and undercarboxylated osteocalcin but not BALP (Kim et al., 2011). Similarly, altering dietary cation–anion balance through anion salts had no effect on BALP (Kurosaki et al., 2007; Schonewille et al., 1994). These findings indicate that BALP and osteocalcin may serve as biomarkers for different aspects of osteoblast activity: BALP is primarily involved in early matrix formation and mineralisation (Stein et al., 1990) whereas osteocalcin binds hydroxyapatite and collagen via osteopontin, contributing to bone structural organisation (Fortune et al., 1989; Gundberg et al., 2002; Hoang et al., 2003; Ritter et al., 1992). Overall, BALP serves as a useful biomarker connecting endocrine–nutritional regulation with bone metabolism. BALP and osteocalcin provide complementary information: BALP reflects early osteoblast activity related to matrix formation and mineralisation, whereas osteocalcin indicates later stages of bone maturation and structural organisation. Together, these markers offer a fuller picture of bone metabolism than either biomarker measured alone.

Growth factors also play a pivotal role affecting BALP levels. Insulin-like growth factor-1 (IGF-1) stimulated BALP production in osteoblasts derived from Holstein cattle reinforcing the strong link between anabolic signalling and bone formation (Li et al., 2009). Collectively, the above findings establish BALP as a sensitive biomarker of skeletal metabolism that is responsive to life stage (young versus mature animals), nutrition, and endocrine regulation.

### Placental-associated ALP activity

PLAP has a critical role particularly in late pregnancy. In Kankrej cattle, PLAP activity rises markedly in mid- and late-pregnancy and facilitates phosphate transfer to support the rapid foetal growth and development that occurs at this stage (Singh et al., 2019). PLAP expression may have diagnostic utility in veterinary oncology. The immunohistochemical detection of PLAP in embryonal carcinomas aids in distinguishing these neoplasms from other tumour types such as yolk sac tumours and mesotheliomas (Aihara et al., 2011). Thus, PLAP represents both a physiological marker of pregnancy and a diagnostic tool in pathology.

### Intestinal-associated ALP activity

IALP has a vital role in intestinal health and phosphate metabolism (Fawley & Gourlay, 2016). This ALP isozyme possesses an Asp-SerP-Ala sequence, conserved across mammalian species, around the active serine residue that is essential for catalytic activity (Engström, 1964). The IALP enzyme exhibits considerable heterogeneity, comprising forms with distinct electrophoretic mobilities, substrate specificities, and thermal stabilities, reflecting its adaptability to diverse intestinal conditions (Behal & Centre, 1965). Functional studies established IALP as both a phosphomonoesterase and a pyrophosphatase, thereby directly contributing to phosphate metabolism (Fernley & Bisaz, 1968). Kinetic experiments further demonstrate the enzyme’s latent regulatory potential: Tris buffer can form a reversible complex that enhances activity by activating an auxiliary site (Neumann et al., 1975), although this effect is not physiologically relevant because Tris is not present *in vivo*. Rather, the importance of such findings lies in showing that IALP possesses allosteric features that can be modulated by small molecules. Base-catalysed reactions across diverse buffer systems confirmed that IALP maintains catalytic efficiency across a broad pH range without undergoing major conformational changes (Bannister & Foster, 1980). In contrast to the *in vitro* Tris effect, divalent cations such as magnesium have a clear biological role: they stabilise IALP’s conformation, enabling refolding and reactivation under stress conditions such as pH fluctuations or thermal shifts, a feature that distinguishes mammalian ALPs from other phosphatases (Tian et al., 2003). Together, these biochemical insights highlight that catalytic efficiency is not just an enzymatic characteristic but a key determinant of IALP’s physiological role in maintaining phosphate homeostasis and protecting intestinal barrier integrity under the dynamic conditions of the bovine intestine. Genetic characterisation of bovine IALP has also identified residues that determine catalytic efficiency, further underscoring its functional adaptability (Manes et al., 1998). Clinically, IALP is particularly relevant in neonatal calves, where elevated levels are associated with intestinal injury or compromised gut integrity, supporting its potential as a biomarker of gastrointestinal health (Besman & Coleman, 1985; Hua et al., 1986; Ok et al., 2020).

### Kidney-associated ALP activity

The kidney regulates phosphorus homeostasis by renal tubular reabsorption. Kidney associated ALP (KALP) contributes to phosphate regulation and to renal metabolism, supporting overall mineral balance. Its biological role is closely tied to the kidney’s capacity to manage phosphate excretion and reabsorption in response to metabolic demands that affect phosphorus homeostasis, such as shifts in phosphorus intake (i.e., low-quality forages), rapid skeletal growth or lactation. Biochemical studies demonstrated that magnesium ions enhance KALP activity through allosteric activation, reflecting its adaptability to metabolic demands, consistent with an enzyme poised to increase dephosphorylation when needed (Cathala and Brunel, 1975a; Cathala & Brunel, 1975). Electrophoretic analysis revealed multiple KALP forms with antigenic similarities to ALPs associated with other tissues, complicating their differentiation in clinical diagnostics (Cavanagh et al., 1981). However, more recent work using modified agarose gel electrophoresis with protease and neuraminidase treatment has successfully separated bovine ALPs fractions (hepatic, bone isoforms and intestinal isozyme were separated), demonstrating that isozymes and isoforms can be resolved and measured in bovine serum (Chiba et al., 2020b). The bovine KALP is the kidney associated isoform of TNAP encoded by the *ALPL* gene. Early molecular work, such as cloning and sequencing kidney-derived ALPs cDNA, showed 90% homology in amino-acid identity with human ALPs (Garattini et al., 1987). This level of conservation is typical of enzymes that are essential to mineral metabolism in most mammals. Further studies are necessary to ascertain which specific transcripts and isoforms are translated into KALP: additional molecular knowledge may help to develop methods for measuring KALP, distinguished from other ALPs.

### Analytical approaches for ALP isozymes and isoforms differentiation

Analytical techniques have been developed to distinguish ALPs isozymes and isoforms, although their application in cattle remains limited. Electrophoretic methods, including agarose gel electrophoresis, allow separation of ALPs based on differences in charge and mobility and have been successfully applied to distinguish hepatic, bone, and intestinal fractions in bovine serum (Chiba et al., 2020b). Heat inactivation profiles provide a simpler approach, exploiting differences in thermal stability between different ALPs, particularly the relative heat resistance of placental-associated ALP compared to liver-associated ALP and BALP (Makris et al., 2023; Moss, 1982). Immunoassays, including BALP assays, offer greater specificity and have been used in studies of bone metabolism (Mohebbi et al., 2010). More recently, proteomic and mass spectrometry-based approaches have enabled precise quantification of peptides associated with specific ALPs and may therefore assist in distinguishing isozymes and isoforms, although these methods remain largely confined to research settings. Further exploration of these modern analytical approaches may support the development of more specific assays for individual ALPs isozymes and isoforms in cattle.

### Analysis of ALPs across cattle life stages

As many studies do not clearly distinguish between isoforms, the following sections discuss ALPs in general. When possible, specific isoforms were referenced.

### Neonatal to adult transition

ALP enzyme activity in cattle follows a distinct developmental trajectory that reflects the physiological demands of each life stage. In neonatal calves, ALPs levels are markedly elevated, supporting rapid skeletal growth and organ development. A study of dairy/beef crossbred calves showed that ALPs peaks shortly after birth at approximately 800 U/L, then gradually declines to around 400 U/L by 83 days of age (Knowles et al., 2000). Both levels reported in young animals are still well above the adult reference range of 90–170 U/L (Animal Health Laboratory, University of Guelph, 2025; Cornell University College of Veterinary Medicine, Animal Health Diagnostic Centre, 2017). The latter finding and subsequent reports highlight the necessity of age-specific reference intervals and isoform-specific assessments when interpreting ALPs in growing cattle (Mohri et al., 2007). A limitation of many studies is the reporting of total ALPs rather than focusing on BALP in bovine growth studies or on LALP when investigating hepatic conditions.

Breed- and parity-specific differences further illustrate the complexity of neonatal ALPs dynamics. For example, Shorthorn calves exhibit an average activity of ALPs 430 U/L on the first day of life (Doornenbal et al., 1988). Milk-fed Simmental calves reach around 420 U/L by the seventh day (Egli & Blum, 1998). In Canchim-Nelore calves, values differ depending on maternal parity: 307 U/L in calves born to primiparous cows compared with 351 U/L in those born to multiparous cows, reflecting differences in early developmental demands and/or differences in colostrum composition between different calf groups (Rocha et al., 2012). Hence, the development of ALPs as a biomarker should consider age and breed differences. Future work could expand on the current literature to include beef cattle breeds, especially *Bos indicus-*derived breeds, which may differ from *Bos taurus*. In addition, inconsistencies across studies may arise from methodological differences, including assay techniques, lack of isozyme and isoform differentiation, and variation in sampling protocols across physiological stages.

Colostrum intake is a significant determinant of postnatal activity of ALPs as substantial amounts of BALP are transferred into the neonatal circulation. This transfer supports skeletal development and may stimulate osteoclastogenesis (Chiba et al., 2020a; Hatate et al., 2019). Calves born to primiparous cows exhibit higher ALPs levels after colostrum ingestion (Rocha et al., 2012), which likely reflects higher BALP levels in younger cows as described in the above section. The timing of colostrum feeding further modulates enzymatic activity: early intake leads to a faster but lower peak, whereas delayed intake produces a slower but higher peak in ALPs levels (Kurz & Willett, 1991; Zanker et al., 2001). These differences in ALPs levels underscore the critical role of colostrum in metabolic and enzymatic maturation during the bovine neonatal period.

As cattle transition from the neonatal stage, ALPs levels decrease, indicating a shift from rapid to slower, yet still marked, growth. In young pubertal beef bulls (approx. 11 months of age), elevated ALPs levels are associated with rapid skeletal growth, whereas lower levels indicate improved feed efficiency (Bourgon et al., 2017). ALPs levels were also influenced by stressful events such as transportation followed by slaughter (Bourgon et al., 2017). The decline in ALPs levels with age in bulls reflects reduced skeletal growth, reduced bone turnover, and perhaps reduced metabolic activity, which may contribute to the onset of osteoporotic changes in older bulls (Forslund et al., 1980).

In young heifers, ALPs levels are a key indicator of metabolic activity, particularly during oestrus. During oestrus, the increase in estrogen influences bone turnover, which is reflected in variations in ALPs levels (Crane et al., 2016). As heifers mature, ALPs levels gradually decrease, reflecting a reduction in the rapid skeletal growth seen at younger ages. Reference values for ALPs levels in dairy cattle can be used to monitor the expected decline in ALPs as heifers age. Specifically, the ALPs mean for heifers is 156.8 U/L, with reference limits ranging from 85.0 to 289.3 U/L, and this is higher than the adult reference range (Brscic et al., 2015). The reduction in ALPs levels in heifers transitioning to adulthood closely aligns with their skeletal growth and reproductive cycle, as ALPs levels modulate phosphorus metabolism in response to bone mineralisation and the cell signalling demands of different life stages (Jonsson et al., 2013).

Elevated ALPs levels during the pre-puberty underscore the importance of this enzyme in supporting rapid growth and development. As cattle transition into the post-puberty reproductive stage, the reduction in ALPs levels aligns with the stabilisation of growth and the shift toward reproductive and maintenance functions. Mostly, studies in mature dairy cows converged to the now established range of 27–127 U/L as being clinically normal (Cornell University Animal Health Diagnostic Center [AHDC], 2025; Doornenbal et al., 1988; Mohri et al., 2007). However, reference levels for each specific isozyme and isoform are not clearly established for each age group or in each cattle breed: a major limitation of current literature. Another age group not covered by current literature is aging animals, ALPs levels in cattle during senescence have not been investigated.

### Lactation and pregnancy

ALP changes during pregnancy and lactation reflect the balance between skeletal metabolism, mineral homeostasis, and the physiological demands of milk production. A progressive rise has been reported from early to late pregnancy, which is reflective of the cumulative metabolic burden as cows prepare for milk production (Brscic et al., 2015). Around calving, BALP concentrations typically decline in both primiparous and multiparous cows due to the combined stress of parturition and the high calcium demand of lactation (Kim et al., 2010; Kurosaki et al., 2007). During early lactation, bone resorption predominates without a corresponding increase in bone formation, which maintains relatively stable BALP activity (Filipović et al., 2008). Changes to bone resorption and formation underscore how cows adapt to the burden of lactation while keeping enzyme activity and circulating phosphorus levels within physiological limits without signs of abnormal hepatic or bone function (Oliveira et al., 2020). The lactation demand in dairy cows is markedly different from the “lighter burden” in beef cows, and further investigation is needed to establish the ranges of normal BALP in the transition period of different cattle breeds, especially *Bos indicus* breeds, which were less prominent in the literature. If ALPs or BALP could be proposed as a biomarker associated with milk fever and other transition period issues, then clear reference values are needed.

Several studies reported that cows exhibit higher ALPs levels during their first lactation, which subsequently stabilise in multiparous animals (Cattaneoet al., 2023; Meyer & Harvey, 2004; Rocha et al., 2019; van Mosel & Corlett, 1990). This parity effect indicates that the first lactation is a critical period of metabolic adjustment, whereas later lactations reflect reduced osteoblastic activity. Parity effects seem logical when considering that primiparous cows might still be growing (demanding calcium for skeletal growth) while mature cows have completed their skeletal growth.

To summarise, ALPs levels in dairy cows are determined by an interplay between parity, lactation stage, and reproductive status. While these intrinsic factors establish baseline activity, extrinsic influences such as nutrition, environmental stressors, and health status can further modulate ALPs levels. Such variability contributes to heterogeneity across studies, as differences in analytical platforms, sampling timing relative to lactation stage, and inconsistent reporting of isozymes and isoforms can substantially influence reported ALPs values.

### ALP in metabolic disorders

Metabolic disorders are associated with alterations in ALPs activity, particularly in conditions affecting energy balance and mineral homeostasis. Increased ALPs levels have been reported in cows with ketosis, reflecting hepatic stress linked to negative energy balance (Shi et al., 2021). Similarly, subclinical hypocalcaemia is associated with elevated ALP, likely due to altered calcium–phosphate metabolism and compensatory bone turnover (Xu et al., 2021). These findings support the role of ALP, particularly bone and hepatic isoforms, as indicators of metabolic adaptation during periods of physiological stress.

### ALP in reproductive disorders

Reproductive conditions also influence activity of ALPs through inflammatory and tissue remodelling processes. Elevated ALPs levels have been observed in cows with retained placenta, suggesting inflammatory responses and delayed uterine recovery (Li et al., 2021). In pyometra, elevated ALPs levels are consistent with systemic inflammation and uterine tissue damage (Amin et al., 2021). These observations indicate that ALPs alterations in reproductive disorders are primarily secondary to inflammatory and repair mechanisms rather than direct metabolic dysfunction.

### ALP in infectious and inflammatory diseases

Infectious diseases induce changes in ALPs activity, often reflecting organ damage and systemic inflammatory responses. Increased ALPs levels in Theileria and Babesia infections are associated with hepatic injury and tissue necrosis, whereas decreased activity of ALPs in *Trichostrongylus* infections suggests intestinal damage (Shayo et al., 1979; Dede et al., 2014; Ghimire et al., 2022; Sandhu et al., 1998). These findings highlight the potential of ALPs as a non-specific biomarker of infection-related tissue damage, although interpretation is limited by the lack of isozyme and isoform resolution in most studies.

### ALP in nutritional and environmental modulation

Nutritional and environmental factors are important modulators of ALPs activity, influencing both metabolic function and physiological adaptation. Dietary interventions have been shown to alter ALPs levels: pumpkin seed protein isolate reduces ALPs activity, suggesting hepatoprotective effects (Li et al., 2021), whereas zinc supplementation increases activity of ALPs and improves rumen function (Wang et al., 2021). Hypozincemia in neonatal calves is associated with reduced ALPs activity, reinforcing its sensitivity to mineral status (El-Maghraby & Mahmoud, 2021).

Environmental stressors, particularly heat stress, have been shown to reduce ALPs levels (Calamari et al., 2018; Tuyttens et al., 2015). However, a controlled study in lactating Holstein cows under moderate to severe heat stress (THI > 80) reported no significant change in total activity of ALPs (p = 0.653) (Blond et al., 2024). Nutritional strategies, including calcium, zinc, and vitamin D supplementation, may mitigate these effects, whereas certain dietary components such as fish oil may increase ALP, potentially reflecting hepatic stress (Kargar et al., 2015; Nasrollahi et al., 2019). However, in our literature search, we did not identify any studies that specifically evaluated ALPs responses in cattle under tropical or subtropical climatic conditions.

### Sources of heterogeneity in ALPs studies

Although the direction of ALPs change is generally consistent across studies (e.g., increased in most infections, decreased under heat stress), the magnitude differs considerably. We identified several sources of this variation. Assay methods and units differ between studies, making direct comparisons difficult (see Table 3). Breed and age also contribute, with neonatal calves have much higher levels of ALPs than adults (Table 4). Sampling protocols are not uniform: tissue sampled, timing relative to calving, lactation, oestrus, or colostrum feeding, all affect reported results. Management factors like shade or diet modify the effect of heat stress as well. These sources of heterogeneity in ALPs studies are additional and overlapping with the infrequent use of specific assays to differentiate isozymes or isoforms. For example, only a few studies have specifically measured BALP (Table 4).

### Future areas for ALPs research in cattle

Future research should prioritise the development of isozyme- and isoform- specific assays for cattle, followed by the establishment of age-, breed-, and production system-specific reference intervals for each ALP. To date, most studies have focused on dairy cattle, leaving important gaps in understanding activity of ALPs across beef production systems and breeds, particularly lacking is ALPs information on *Bos indicus* cattle (Table 4). Longitudinal, multi-breed cohorts are needed to quantify how diet (e.g., mineral and vitamin strategies) and environmental factors (e.g., heat load and parasites) influence ALPs measurements. Expanding reference values across breeds and production systems would further support the veterinary application of ALPs.

Genetic influences on ALPs measurements also remain underexplored. The annotation of ALP-related genes in the bovine reference genome (Table 5), including *ALPL* and *ALPI*, and associated regulatory regions, provides a foundation for understanding isozyme and isoform differences in terms of expression and function. Comparative and population genomics approaches may help link genetic variation with isozyme and isoform activity that may underpin phenotypic differences between *Bos taurus* and *Bos indicus* cattle. However, variation in circulating activity of ALPs is unlikely to reflect simple one gene–one phenotype relationships, as ALPs measurements are influenced by multiple genetic and non-genetic factors. Although ALP-related phenotypes may be biologically closer to the underlying genotype than complex production traits, their genetic architecture remains poorly understood.

The differentiation of ALPs isozymes and isoforms has important diagnostic implications. Total activity of ALPslacks specificity, as changes may originate from bone, liver, or other tissues. In contrast, isoform- and isozyme-specific measurements allow more precise interpretation of physiological and pathological processes. For example, BALP reflects osteoblastic activity and skeletal growth, whereas hepatic ALP is more indicative of hepatobiliary function or metabolic stress. The limited use of isozyme and isoform differentiation in bovine studies restricts the clinical applicability of ALPs measurements and likely contributes to inconsistencies across the literature. Expanding isoform- and isozyme-specific assays is therefore essential to improve the diagnostic value of ALPs in cattle.

In breeding programs, specific ALP isozymes or isoform profiles may prove useful as selection-adjacent phenotypes for traits related to skeletal growth, mineral metabolism, liver function, or resilience to metabolic stress. In herd management, establishing age-, breed-, and isozyme-and isoform-specific reference intervals, particularly for beef cattle, would support the integration of ALPs into routine monitoring and improve the distinction between adaptive physiological responses and pathological conditions. Overall, integrating isozyme- and isoform- resolved phenotyping with genomic information may enhance the utility of ALPs as biomarkers for cattle breeding, nutrition, and welfare diagnostics.

Studies in cattle suggest an association between IALP and intestinal injury. However, mechanistic understanding remains limited in cattle compared with the growing body of human literature on intestinal barrier dysfunction (Singh et al., 2022). Thus, highlighting another important area for future bovine research.

## Conclusion

This bibliometric and narrative review has shown that the activity of ALPs, primarily in dairy cattle, is extensively studied across various physiological and pathological conditions including metabolic adaptation during lactation, bone health, liver function, and responses to dietary supplements and diseases. The literature revised consistently supports biologically meaningful associations between activity of ALPs and cattle health or metabolic status. However, the clinical and diagnostic application of ALPs remains limited by assay variability, the biological non-specificity of total ALP, and the lack of standardised reference intervals, including condition-specific thresholds across breeds, ages, and physiological stages. This review provides a knowledge platform that informs future research. Further studies on ALPs isozymes and isoforms, across multiple cattle breeds, and especially *Bos indicus* cattle, are needed to fully elucidate the roles of ALPs and to establish detailed reference levels that may support future biomarker development, cattle management and breeding strategies, and veterinary diagnostics.

## Funding sources

This research received no specific grant from funding agencies in the public, commercial, or not-for-profit sectors.

## Ethical statement

This systematic review relied on published data, no new data and no new samples were collected, and as such no animal ethics approval was required.

## CRediT authorship contribution statement

**Daniya Smagulova:** Writing – original draft, Methodology, Investigation, Formal analysis. **Charlotte Dawson:** Writing – review & editing, Investigation. **Guilherme de Padua Nogueira:** Writing – review & editing, Supervision. **Michael J. D’Occhio:** Writing – review & editing, Supervision. **Laercio R. Porto-Neto:** Writing – review & editing, Conceptualization. **Isabella C. de Castro Lipp:** Writing – review & editing, Visualization, Investigation. **Marina R.S. Fortes:** Writing – review & editing, Supervision, Investigation, Conceptualization.

## Declaration of competing interest

The authors declare that they have no known competing financial interests or personal relationships that could have appeared to influence the work reported in this paper.
